# Supplementary material for: Perceptions of Light Pollution and its Impacts: Results of an Irish Citizen Science Survey
Source: Int J Environ Res Public Health. 2020 Aug 4;17(15):5628. doi: 10.3390/ijerph17155628 (PMC7432530; doi:10.3390/ijerph17155628)
Supplement: Supplementary file 1 [file ijerph-17-05628-s001.pdf]

ALAN Questionnaire

Light at Night Questionnaire

Q.1
Which of the following best describes where you live?

- Rural area
- Small town (<3,000 people)
- Large town (>3,000 people)
- City Centre
- City Suburbs
- Other:

Q.2
How bright is the night-time environment around where you live (or work, if you are a night worker)?

- Completely dark (Milky Way regularly visible to the horizon)
- Dark (>100 stars and Milky Way regularly visible overhead)
- Few stars visible and no Milky Way
- Only Moon and brighter planets visible
- Unsure

Q.3
What is the strongest light source near your home?

- Own domestic lighting
- Neighbours' lighting
- Commercial/Retail lighting
- Public lighting
- Passing traffic
- Other:

Q.4
Is this strong light source on all night?

- Yes
- No
- Intermittent
- Unsure

Q.5
Please indicate your level of agreement with the following statements

	Strongly Disagree	Disagree	Somewhat Disagree	Neutral	Somewhat Agree	Agree	Strongly Agree	Not Applicable / No Opinion
'The level of lighting near my home at night has increased over the past three years'								
'If light enters my bedroom at night it does not affect my sleep'								
'Birds sing at night (the dawn chorus starts earlier than it used to)'								
'The number of bats I see has increased recently'								
'The natural night-time behaviour of insects/bats/foxes etc., remains the same as in previous years'								

Q.6
Which of the following statements best describes your attitude towards night-themed events (such as dark sky festivals, night walks/runs, etc)?

Please select all that apply

- ☐ I have previously participated in night-themed events
- ☐ I am interested in attending night-themed events in the future
- ☐ I am not interested in night-themed events
- Other: ____

Q.7
What is your age?

- 18-24
- 25-34
- 35-44
- 45-54
- 55-64
- 65+
- Prefer not to say

Q.8
What is your gender identity?

- Male
- Female
- Prefer not to say
- Other:
